# Determination of Drug Efflux Pump Efficiency in Drug-Resistant Bacteria Using MALDI-TOF MS

**DOI:** 10.3390/antibiotics9100639

**Published:** 2020-09-24

**Authors:** Wen-Jung Lu, Hsuan-Ju Lin, Pang-Hung Hsu, Hong-Ting Victor Lin

**Affiliations:** 1 Department of Food Science, National Taiwan Ocean University, No. 2, Pei-Ning Road, Keelung 202, Taiwan; miss350100@gmail.com (W.-J.L.); angel810801@gmail.com (H.-J.L.); 2 Department of Bioscience and Biotechnology, National Taiwan Ocean University, No. 2, Pei-Ning Road, Keelung 202, Taiwan; 3 Center of Excellence for the Oceans, National Taiwan Ocean University, No. 2, Pei-Ning Road, Keelung 202, Taiwan; 4 Institute of Biochemistry and Molecular Biology, National Yang Ming University, No. 155, Sec. 2, Linong Street, Taipei 112, Taiwan

**Keywords:** AcrB, drug resistance, efflux activity, MALDI-TOF MS, multidrug transporter

## Abstract

Multidrug efflux pumps play an essential role in antibiotic resistance. The conventional methods, including minimum inhibitory concentration and fluorescent assays, to monitor transporter efflux activity might have some drawbacks, such as indirect evidence or interference from color molecules. In this study, MALDI-TOF MS use was explored for monitoring drug efflux by a multidrug transporter, and the results were compared for validation with the data from conventional methods. Minimum inhibitory concentration was used first to evaluate the activity of *Escherichia coli* drug transporter AcrB, and this analysis showed that the *E. coli* overexpressing AcrB exhibited elevated resistance to various antibiotics and dyes. Fluorescence-based studies indicated that AcrB in *E. coli* could decrease the accumulation of intracellular dyes and display various efflux rate constants for different dyes, suggesting AcrB’s efflux activity. The MALDI-TOF MS analysis parameters were optimized to maintain a detection accuracy for AcrB’s substrates; furthermore, the MS data showed that *E. coli* overexpressing AcrB led to increased ions abundancy of various dyes and drugs in the extracellular space at different rates over time, illustrating continuous substrate efflux by AcrB. This study concluded that MALDI-TOF MS is a reliable method that can rapidly determine the drug pump efflux activity for various substrates.

## 1. Introduction

Multidrug transporters are cell membrane glycoproteins that excrete various classes of compounds across cell membranes. Active transport of molecules is one of the most significant mechanisms of drug resistance in bacteria. Multidrug transporters are classified into two categories: ATP binding cassette transporters and secondary active transporters. The drug excretion systems that utilize the free energy from ATP hydrolysis to actively transport substrates across a membrane belong to the ATP binding cassette transporters group [1,2]. Secondary active transporters, on the other hand, are driven by a proton motive force. These transporters have been categorized into several families in terms of their protein sequence similarities: the major facilitator superfamily (MFS), the multidrug and toxic compound extrusion (MATE) family, the small multidrug resistance (SMR) family and the resistance nodulation division (RND) family [3,4]. One of the most studied secondary active transporters is *Escherichia coli* AcrB, which belongs to the RND family and is significant in transporting a wide range of substrates [5].

There are many approaches to measuring the activity of multidrug transporters, and these methods can be ascribed to two categories: (i) susceptibility assays such as the disc diffusion assay and broth dilution method for determination of minimum inhibitory concentration (MIC) and half inhibition concentration (IC_50_) and (ii) assays that evaluate how much substrate is accumulated in the cells, such as the fluorescent accumulation assay and efflux assay, where dyes are used to give differential fluorescence for intracellular and extracellular spaces [6].

In the disc diffusion assay and broth dilution assay, when bacteria overexpress an efflux transporter, they are less susceptible to tested chemical compounds, and this indicates the efflux activity of the transporter [7,8]. The MIC results between transporter deficient and transporter expressing strains have been used extensively as an evaluation method for the function of drug pumps [9,10] and efflux pump inhibitors [11,12,13]. A review by Prof. Piddock’s group [6] described many methods to measure the efflux activity of transporters, including MIC assays. However, minor variations in methodology and manipulation can result in large variations in MIC; for example, prolonged incubation and a smaller inoculum may change the apparent MIC. In addition, the differences in the MICs of drugs when used in drug-resistant cells expressing transporters versus when used in drug-susceptible cells do not necessarily indicate drug translocation, and vice versa; thus, it is hard to conclude that there has been no drug translocation with similar MICs [14].

An accumulation assay has been frequently used to evaluate amounts of substrates accumulated in the cells, and these amounts imply the level of the efflux of substrates. Generally, radiolabeled molecules or fluorescent dyes have been chosen, so their accumulated levels could be determined within the detected cells expressing drug transporters [15]. Dyes such as ethidium bromide (EtBr) [16] and Hoechst 33342 [17] show stronger fluorescence when bound to DNA and/or in a hydrophobic environment. Another approach to studying the efflux capacity of transporters is to incubate cells with preloaded dyes that diffuse into the cells, where the addition of glucose can reenergize the cells so that transporters will extrude the dyes [14,18]. The efflux assay could directly provide a real-time efflux curve of the dye mediated by transporters, data that cannot be determined from MIC results [19]. Although such an assay is convenient, it sometimes does not distinguish well among cells that have slightly different efflux activities by using fluorescence detection [20]. In addition, self-quenching of fluorescence signals from H33342 and EtBr has been reported at higher levels inside the cells [21,22]. Furthermore, use of fluorescent detection for screen compounds or even complex samples for inhibitory activity against drug transporters could pose problems, because some molecules might quench or contribute to the fluorescent signal; for example, flavonoid quercetin and polyphenols have been reported to quench the fluorescence of EtBr in fluorescent studies [23,24].

Matrix-assisted laser desorption ionization–time of flight mass spectrometry (MALDI-TOF MS) is one of the most popular MS techniques in routine laboratory research and clinical microbiology due to its speed, economy and diagnostic benefits [25]. However, its application in the detection of substrate efflux by drug transporters has not been elucidated. The goal of this study is to establish a rapid, reliable method using MALDI-TOF MS to monitor dye and drug efflux by drug transporters, and the secondary drug transporter AcrB (RND family) from *E*. *coli* [26] was chosen as the efflux model to measure the efflux of dyes (EtBr, Hoechst 33342 and Nile red) and drugs (erythromycin and rifampicin) over time.

## 2. Results and Discussion

### 2.1. Efflux Pump Modulator PAβN and CCCP Decrease the IC_50_ against Drugs and Dyes in E. coli

AcrB, the most studied transporter in the RND family, is a trimeric inner membrane transporter that confers resistance to a wide range of antibiotics (e.g., tetracycline, chloramphenicol, β-lactams, novobiocin, fusidic acid, nalidixic acid and fluoroquinolones), detergents (e.g., sodium dodecyl sulfate [SDS] and Triton X-100), various cationic dyes, disinfectants and even solvents [27]. The pSYC-acrB expression construct was transformed into the *E*. *coli* strain Kam3 (Δ*acrB*) for protein production and for drug susceptibility testing. The efficiency of expression, purification and identification of AcrB was determined by SDS-polyacrylamide gel electrophoresis (Supplementary Figure S1A). The migration of the AcrB protein, which has 1049 amino acid residues with a predicted molecular mass of approximately 113 kDa, was between 93 and 125 kDa (Supplementary Figure S1A, lane E). Further identification of AcrB was accomplished using LC-MS/MS, with 41% sequence coverage (Supplementary Figure S1B). These data indicated that AcrB protein was successfully overexpressed in *E*. *coli* Kam3. As shown in Supplementary Table S1, *E*. *coli* Kam3 overexpressing AcrB presented increased resistance against macrolide drugs, including erythromycin and clarithromycin 32- and 8-fold, respectively, when compared with the Kam3 harboring an empty plasmid (Kam3).

Carbonyl cyanide *m*-chlorophenyl hydrazone (CCCP) is a protonophore and causes dissipation of the membrane potential, affecting all secondary transporter-dependent effluxes [28], and the peptidomimetic compound phenylalanine-arginine β-naphthylamide (PAβN) has been shown to be an RND pump inhibitor [29]. Overexpression of AcrB confers resistance against various drugs and dyes in *E*. *coli*, and the effects of PAβN and CCCP on AcrB conferring resistance can be evaluated by the IC_50_. As shown in Table 1, resistance against the macrolide drugs erythromycin and clarithromycin was reduced eight- and four-fold (modulation factor, MF) in Kam3 cells overexpressing AcrB in the presence of the RND transporter inhibitor PAβN. Moreover, addition of PAβN reduced the IC_50_ of tetracycline, Hoechst 33342 and EtBr two-fold but showed no effects on the IC_50_ of norfloxacin when compared with that of the control group (no addition of PAβN or CCCP). Intriguingly, addition of PAβN greatly reduced the IC_50_ to 0.019 μg/mL against the semisynthetic antibiotic rifampicin in Kam3-AcrB, and this IC_50_ for rifampicin was even lower than that for Kam3 (data not shown), suggesting that there is an additional mechanism of action for PAβN. A similar result has been reported by Opperman et al. [30], such that the MIC shift produced by PAβN for rifampicin was greater than that for the *acrB* deletion strain. PAβN did not seem to potentiate the activity of Norfloxacin against Kam3-AcrB, and similar results were observed in other studies. Opperman et al. [30] have reported an increasing MIC of norfloxacin with the addition of PAβN in *E. coli* strain AB1157. In addition, Soto et al. [31] indicated that no modulation activity of PAβN was observed for norfloxacin in *Salmonella* Enteritidis isolates expressing AcrB-like efflux transporter. Previous docking studies indicated that norfloxacin could bind to the CH3 site of AcrB [32], and PAβN could bind to the distal binding pocket (DBP) of AcrB [33]. Nakashima et al. [34] indicated that erythromycin/rifampicin bound to the proximal binding pocket (PBP) of AcrB and concluded that a glycine-rich swinging loop located between the proximal binding pocket and distal binding pocket acts as a swinging valve during the drug’s translocation by peristaltic motion [34], which might suggest that competitive inhibitor PAβN could possess a better inhibitory effect on the efflux of PBP-binding substrates than the efflux of CH3-binding substrates. The protonophore CCCP reduced the IC_50_ against the macrolide drugs erythromycin and clarithromycin in Kam3-AcrB 8- and 25-fold. In addition, CCCP could also decrease the IC_50_ for norfloxacin (100-fold), tetracycline (2-fold), H33342 (32-fold) and EtBr (4-fold). Intriguingly, CCCP failed to reduce the IC_50_ for action against rifampicin, a broad-spectrum antibiotic that inhibits DNA synthesis for the treatment of bacterial infections such as tuberculosis. Although the reasons were not determined, such a phenomenon has been observed in some other studies. Li et al. [35] investigated the effects of the efflux inhibitors, such as verapamil, thioridazine, chlorpromazine and CCCP, in terms of rifampicin resistance in *Mycobacterium tuberculosis* clinical isolates. The channel blockers verapamil and chlorpromazine exhibited stronger potentiating activity for rifampicin, whereas CCCP failed to lower or even increase the resistance against rifampicin in some clinical isolates. To sum up, Table 1 provided the modulation activity of PAβN and CCCP for drug erythromycin/rifampicin and dye H33342/EtBr/Nile red against Kam3-AcrB, and we will further explore the efflux of these substrates using fluorescent studies and MALDI-TOF in the following sections.

### 2.2. Dye Accumulation Reduced by Efflux Pump Modulator PAβN and CCCP in E. coli

**Figure 1:**
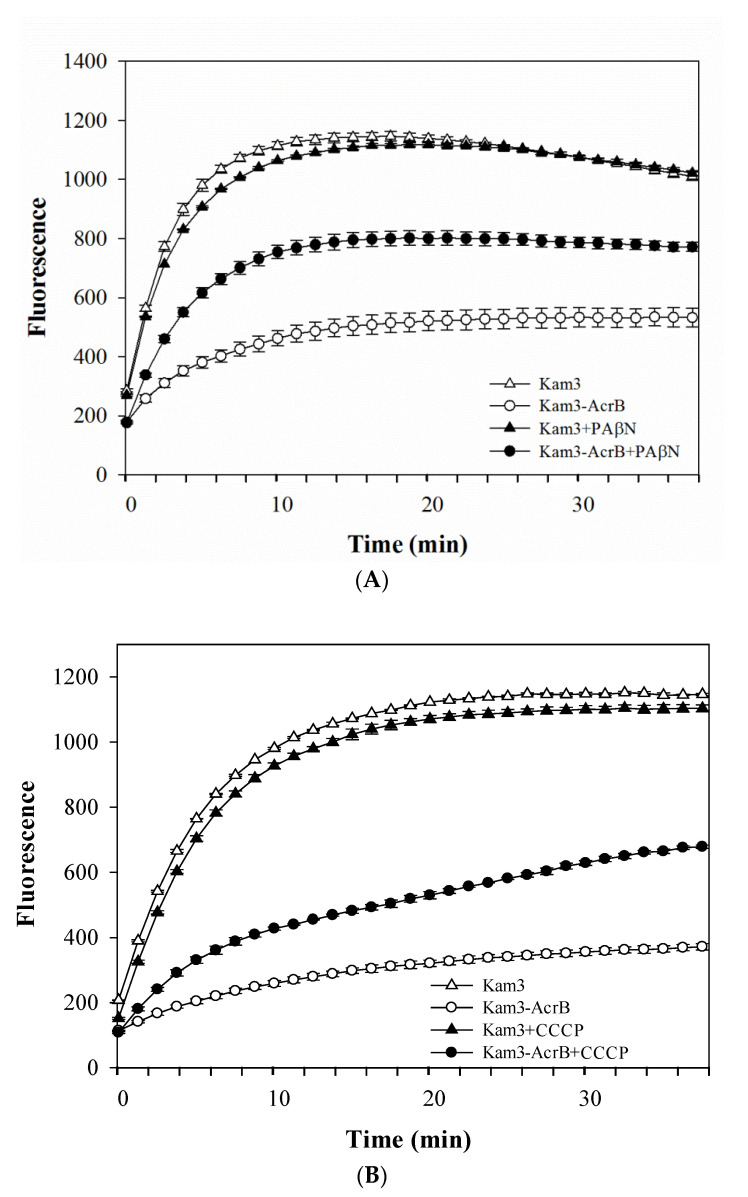
H33342 accumulation in *E. coli* Kam3-AcrB and Kam3 in the presence of efflux pump inhibitor (**A**) PAβN, (**B**) CCCP. The fluorescence was recorded at excitation and emission wavelengths 360 and 460 nm, respectively, over 38 min of incubation time. Values are expressed as mean ± standard deviation (SD) (*n* = 3).

**Figure 2:**
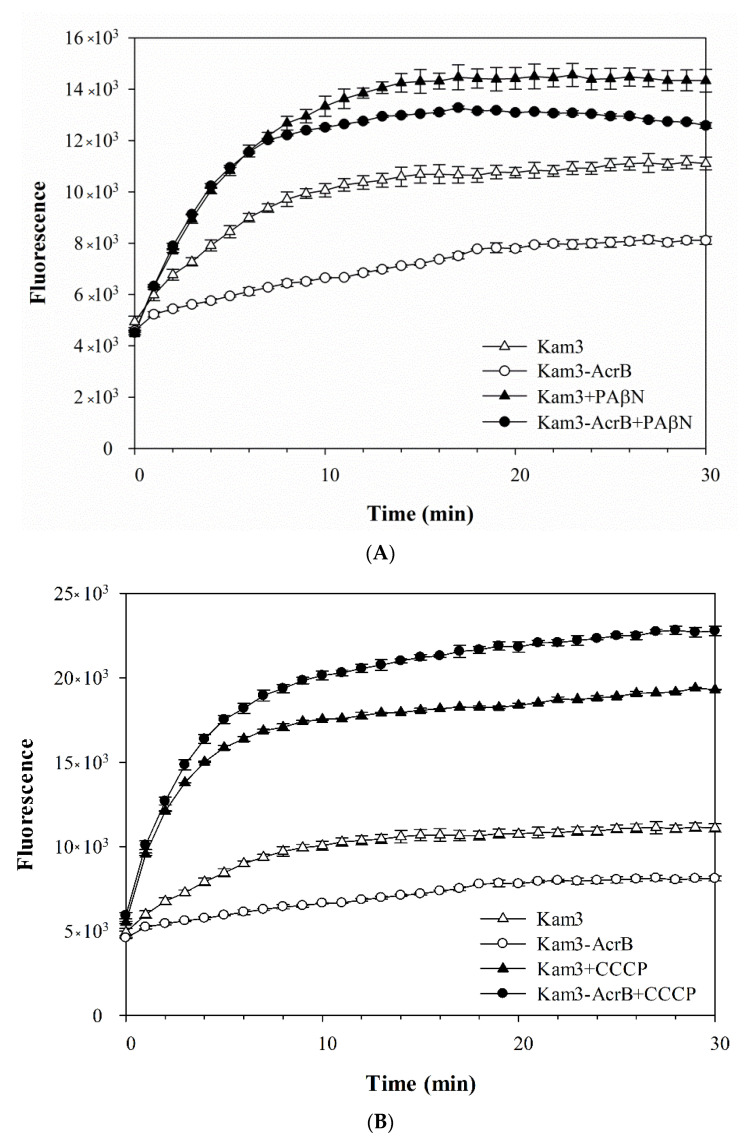
EtBr accumulation in *E. coli* Kam3-AcrB and Kam3 in the presence of efflux pump inhibitor (**A**) PAβN, (**B**) CCCP. The fluorescence was recorded at excitation and emission wavelengths 520 and 600 nm, respectively, over a 30 min incubation period. Values are expressed as mean ± standard deviation (SD) (*n* = 3).

The efflux of the tested antimicrobial agents could not be determined by using MIC tests, and this study further used dye accumulation for the measure of the possible efflux activity of AcrB. Fluorescent compounds, such as H33342 [36], EtBr [20] and Nile red [37], have been widely used to measure efflux by membrane transporters. H33342 fluoresces with a higher quantum yield when it is bound to DNA or in a hydrophobic environment such as a lipid membrane, and EtBr has more intense fluorescence when intercalating with DNA [38,39]. This phenomenon of fluorescing dyes has enabled discrimination between intracellular and extracellular localization of the dyes. PAβN (also known as MC-207,110) is a well-known efflux pump inhibitor for MexAB, MexCD and MexEF pumps in *P. aeruginosa*, and AcrB pump in *E. coli* [40,41]. As shown in Figure 1A, the accumulation level of H33342, a substrate for AcrB, was higher in Kam3 cells than that in AcrB-overexpressing cells, suggesting that AcrB overexpression could reduce the intracellular accumulation of H33342, possibly indicative of H33342 efflux by AcrB. Addition of PAβN to the Kam3-AcrB increased the H33342 accumulation, which indicates that the H33342 efflux by AcrB was retarded by the RND pump inhibitor PAβN. Similar results can be observed in Figure 1B, where it is shown that the proton uncoupler CCCP increased H33342 accumulation in Kam3-AcrB. EtBr, another dye substrate for AcrB, was also used to measure the efflux efficiency of AcrB. As shown in Figure 2A, a higher level of EtBr accumulation was observed in Kam3 than that in Kam3-AcrB, and addition of PAβN increased the intracellular accumulation of EtBr in Kam3-AcrB. Similar results are also presented in Figure 2B, where CCCP is shown to increase the EtBr accumulation in Kam3-AcrB. These data indicated that AcrB could reduce the intracellular accumulation of H33342 and EtBr, possibly via active efflux by AcrB, which could be inhibited in the presence of the inhibitors PAβN and CCCP.

### 2.3. Pump Efflux Reduced by Efflux Pump Modulator PAβN and CCCP in E. coli

The accumulation data provide only indirect evidence for the efflux activity of AcrB and the modulation effects of efflux pump modulators PAβN and CCCP, because the level of substrate efflux was not measured directly but was inferred from the level of substrate accumulation. In addition, the amounts of the dyes inside the cells might be affected by cell membrane permeability and uptake of substrates, making it difficult to assess differences in substrate efflux between different strains or species [42]. This study further uses efflux assays along with various dyes, such as H33342, EtBr and Nile red, to determine the efflux capacity of AcrB.

**Figure 3:**
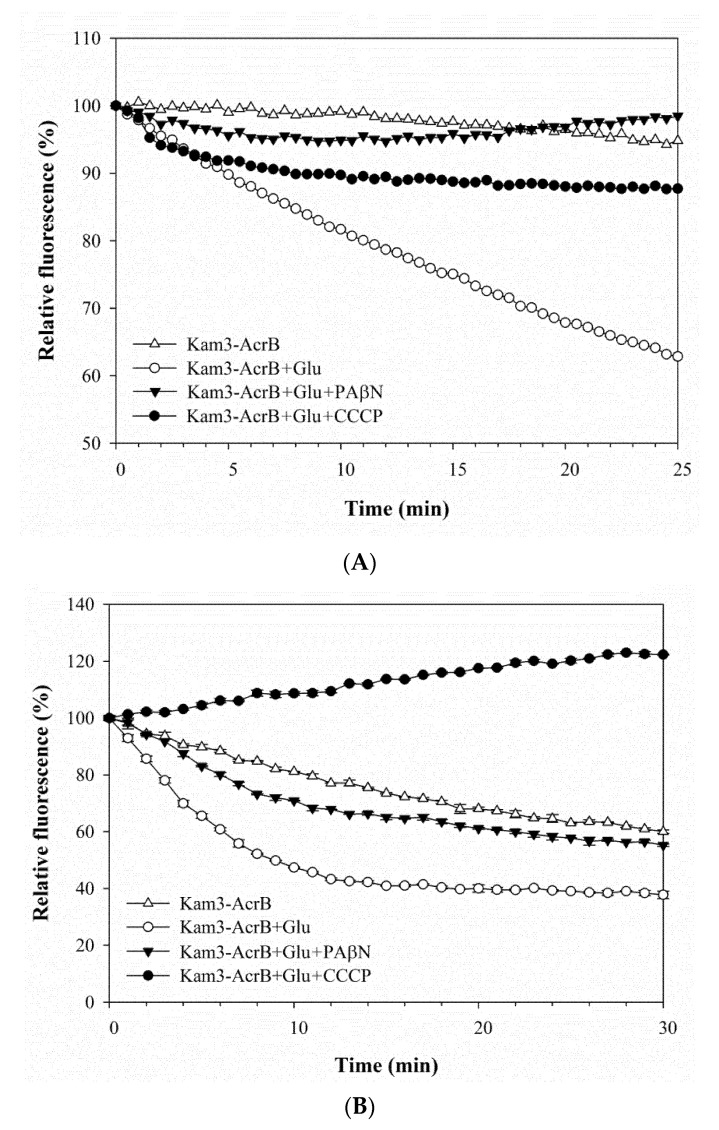

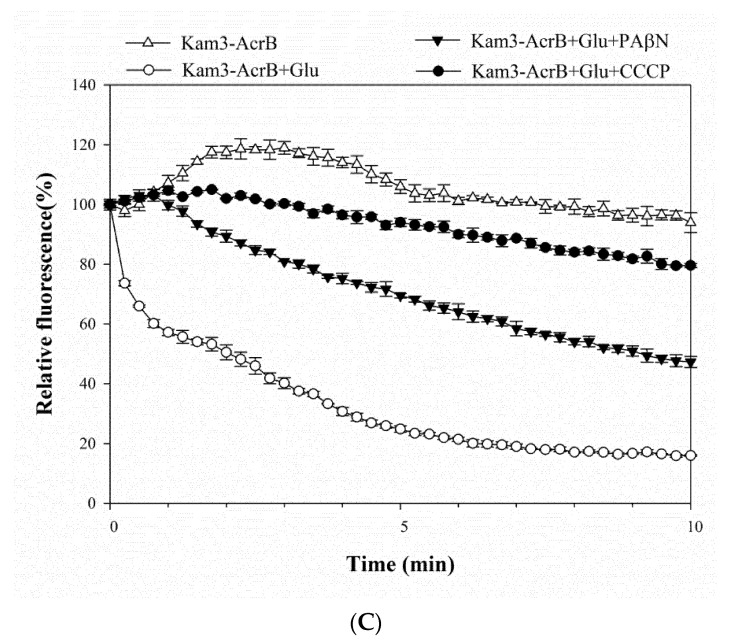
Efflux of dye (**A**) H33342, (**B**) EtBr, (**C**) Nile red in Kam3-AcrB in the presence of glucose and efflux pump inhibitor PAβN and CCCP. The fluorescence was recorded at excitation/emission wavelengths of 360/460, 520/600, 550/636 nm, respectively, over a 10 min incubation period. Glu, glucose. Values are expressed as mean ± standard deviation (SD) (*n* = 3).

As shown in Figure 3A, the addition of glucose to Kam3-AcrB (Kam3-AcrB + Glu) could energize the cells to pump H33342 out of the cells, and a 40% reduction in H33342 fluorescence was observed at 25 min as compared with that of the control. In addition, H33342 efflux was inhibited in the presence of PAβN or CCCP, such that the fluorescence decreased by 2% and 10%, respectively. Greater EtBr efflux was also observed in the cells with added glucose, where EtBr fluorescence was reduced by 60% (Figure 3B). CCCP appeared to totally abolish the efflux of EtBr, and the presence of PAβN reduced the dye fluorescence by 45% (Figure 3B). The fluorescence of Nile red in the control cells increased slightly at the beginning of the measurement and returned to the initial readings later (Figure 3C). Addition of glucose to Kam3-AcrB dropped the fluorescence dramatically to 16% in 10 min, suggesting rapid efflux of Nile red. This might be because lipophilic Nile red remains largely periplasmic and binds to membrane phospholipids, whereas AcrB collects its substrates from the periplasmic space and outer leaflet of the inner membrane. Addition of PAβN or CCCP appeared to retard the Nile red efflux in the cells because the drop in fluorescence was alleviated as compared with that in the Kam3-AcrB + Glu group (Figure 3C).

The fluorescence curves of H33342, EtBr and Nile red in the efflux assays were further investigated for the efflux rate constants (K value) and t_efflux50%_ by fitting the curve into the equation [Y = (Y_0_ − Plateau) × exp(−K × X) + Plateau]. The K value represents the change in the fluorescent intensity over a period of time, and t_efflux50%_ corresponds to the time required for 50% efflux of the preloaded dye. Accordingly, the K value was calculated to be 0.003, 0.18 and 0.36 min^−1^ for the efflux of H33342, EtBr and Nile red, respectively, in the energized Kam3-AcrB. A lower efflux K value leads to greater t_efflux50%_; consequently, the calculated t_efflux50%_ values for the efflux of H33342, EtBr and Nile red were 44.8, 9.29 and 1.87 min in the energized Kam3-AcrB. The K value and t_efflux50%_ were also calculated for the efflux of the dyes in the presence of the efflux pump modulators PAβN and CCCP. The t_efflux50%_ of the efflux of Nile red increased to 9.26 and 21.6 min with the addition of PAβN or CCCP, respectively. The t_efflux50%_ of the H33342 and EtBr efflux could not be calculated with the addition of efflux pump inhibitors, indicating that PAβN and CCCP could abolish the activity of AcrB in the efflux of H33342 and EtBr. In a previous study, EtBr efflux rates were determined in *E*. *coli* AG100, AG100A and AG100_TET_ with K values of 0.0173, 0.0106 and 0.023 min^−1^, respectively. The MIC for EtBr in AG100, AG100A and AG100_TET_ was 150, 5 and 300 μg/mL, indicating that the efflux rates correlated with the MIC for EtBr for each strain [16]. The efflux of Nile red has been optimized for a real-time efflux assay to measure the activity of the AcrAB-TolC system. The addition of the known inhibitors PAβN and NMP inhibited Nile red efflux in *E*. *coli* 3-AG100 and AG102, and the t_efflux50%_ of Nile red efflux was also found to be extended in a dose-dependent manner [43].

This study used the drug transporter AcrB as a model to calculate the K value for the efflux of H33342, EtBr and Nile red; the results show that AcrB exhibits a different rate for efflux of substrates. In addition, the MIC of H33342 and EtBr was decreased in the presence of PAβN and CCCP, and a similar result was presented for t_efflux50%_, showing a correlation between MIC and t_efflux50%_.

### 2.4. Measuring the Dye Efflux by Using MALDI-TOF

The drawback of using fluorescence to monitor pump efflux is that fluorescence can be interfered by heavy metal cations, high concentrations of substrates or the use of a combination of efflux pump inhibitors with strong colors [12,22,43,44]. Berberine is a plant alkaloid and has efflux pump inhibitor (EPI) property against the *S. aureus* NorA, but it was able to intercalate with DNA interfering with the fluorescence of EtBr [45]. Similar phenomenon has been reported by Dos Santos et al. [46] in that phenolic compounds caffeic acid and gallic acid could inhibit the drug efflux in *S. aureus*, and they could interfere with the fluorescent of EtBr by intercalating with DNA.

In order to directly determine the substrate efflux achieved by the drug transporter and to prevent any possible interference, MALDI-TOF MS served to monitor the efflux of the substrates, such as H33342, EtBr and Nile red, in extracellular space. As shown in the Figure S2A, substrates started to move across the cell membrane by passive diffusion upon the addition of the substrates. The influx rate of the substrates was much greater than the efflux rate at the beginning, and the substrates influx gradually reached a plateau (Figure S2A,B) [47]. It has been indicated that chemical properties of antibiotics could affect the rates of diffusion and the final equilibrium distribution across the membrane bilayer [48]. The substrate diffusion rate, the time to reach a substrate influx plateau and the concentration ratio of extracellular and intracellular substrates are largely related to the hydrophobicity of the substrates. Intracellular accumulation of radioactively-labeled norfloxacin in *E. coli* was found to reached a plateau in 2 min, and the amounts of accumulated norfloxacin varied at 4 °C and 25 °C [49]. Cinquin et al. [50] investigated the intracellular accumulation of drug fleroxacin and two topoisomerase inhibitors in *Enterobacter aerogenes* cells and found that the drug influx reached a plateau within 5 min upon addition of the drugs, and the presence of CCCP could increase their intracellular accumulation. Raherison et al. [47] indicated that, in 5 min upon drug addition, 65% of ciprofloxacin and 95% of total pefloxacin (more hydrophobic than ciprofloxacin) were accumulated in the *Mycoplasma hominis*. As shown in Figure S2C, efflux of the substrates by AcrB could be faster than the influx the substrates after influx plateau, causing the increased substrates in the extracellular space, whereas in the presence of EPI, the extracellular substrates did not increase. In this study, MALDI-TOF was used to monitor the extracellular substrates over time, as indicative of pump efflux efficiency.

Optimization of matrix selection, laser energy and the concentration range of substrates is necessary when using MALDI-TOF to monitor substrate efflux. Two common MALDI matrices for small molecule detection, α-cyano-4-hydroxycinnamic acid (CHCA) and 2,5-dihydroxybenzoic acid (DHB), were tested in this study, and these two matrices share a similar pattern of H33342, EtBr and Nile red in the mass spectra. DHB was selected for the detection of substrates due to its better crystal formation and stronger signals under the same laser conditions. The calibration curves of H33342 (1 to 10 ppm), EtBr (0.1 to 10 ppm) and Nile red (0.32 to 80 ppm) were accomplished, where the *r*^2^ values were determined to be 0.9999 for all the calibration curves, representing a good correlation of intensity and dye concentration (Supplementary Figure S3). To maintain MS analysis accuracy, the standard curve for each substrate was freshly prepared and measured for each test.

**Figure 4:**
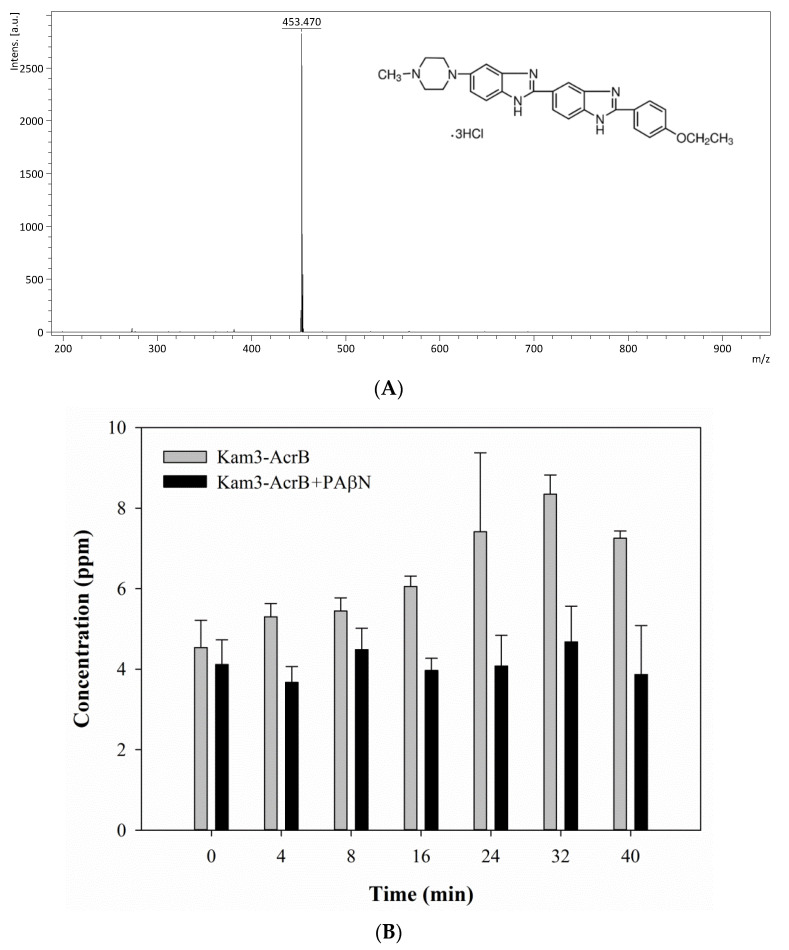
The Hoechst 33342 efflux activity of Kam3-AcrB was detected by using MALDI-TOF MS in the presence of modulator PAβN. (**A**) The mass spectrum of H33342 and (**B**) the intensity of extracellular H33342. The intensity was plotted at the main peak at *m*/*z* 453.47 of H33342 and the detection period was 40 min. Values are expressed as mean ± standard deviation (SD) (*n* = 3).

When the Kam3-AcrB cells were incubated with the substrates, samples were taken from the extracellular space at multiple time points in order to monitor the excreted substrate intensity as a function of incubation time. The mass spectrum of H33342 is shown in Figure 4A and exhibits a main peak at *m*/*z* 453.47, correlating with the loss of three HCl from the H33342 molecule (monoisotopic mass: 560.16 Da). As shown in Figure 4B, the concentration of H33342 in the extracellular space was initially at 4.54 ± 0.68 ppm with the Kam3-AcrB cells, and it reached 8.35 ± 0.48 ppm at 32 min, showing an approximate 1.8-fold increased during 32 min. However, the extracellular intensity of H33342 in the presence of PAβN was maintained at a range of 3.68 to 4.68 ppm, indicating that the efflux activity of AcrB was retarded.

**Figure 5:**
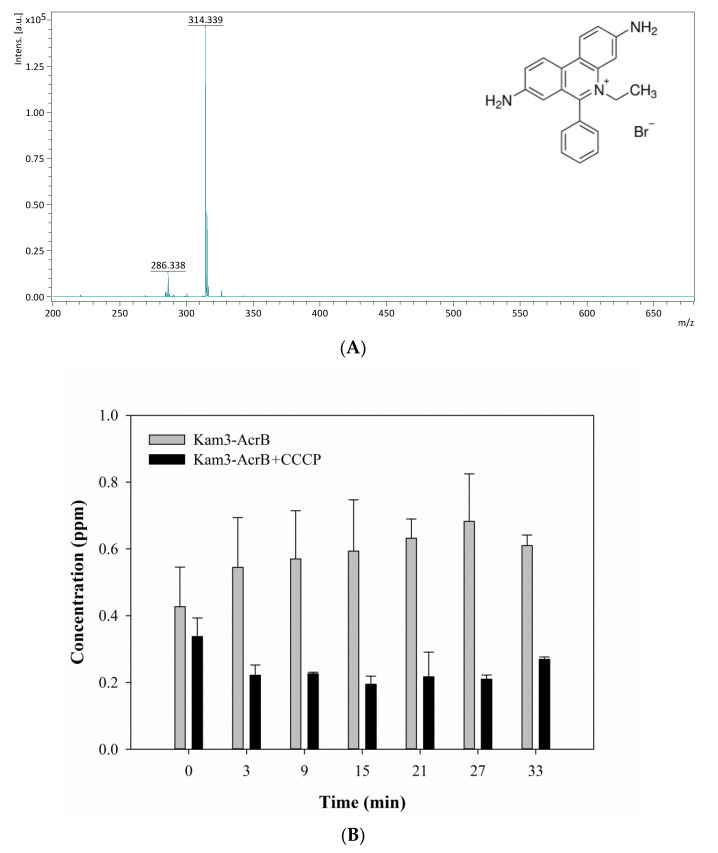
The ethidium bromide efflux activity of Kam3-AcrB was detected by using MALDI-TOF MS in the presence of modulator CCCP. (**A**) The mass spectrum of EtBr and (**B**) the intensity of extracellular EtBr. The intensity was plotted at the main peak at *m*/*z* 314.34 of EtBr and the detection period was 33 min. Values are expressed as mean ± standard deviation (SD) (*n* = 3).

As in Figure 5A, EtBr showed a peak at *m*/*z* 314.34 due to the loss of Br (EtBr: monoisotopic mass 393.08). The efflux of EtBr by AcrB was measured by monitoring the concentration changes of extracellular EtBr over time. The Kam3-AcrB cells in the absence of CCCP increased the EtBr concentration from 0.43 ± 0.12 (*t* = 0) to 0.68 ± 0.14 ppm at 27 min in the extracellular space. Not surprisingly, over time, in the culture medium, decreased EtBr intensity was observed in the presence of CCCP, indicating the ability of CCCP to inhibit AcrB (Figure 5B).

**Figure 6:**
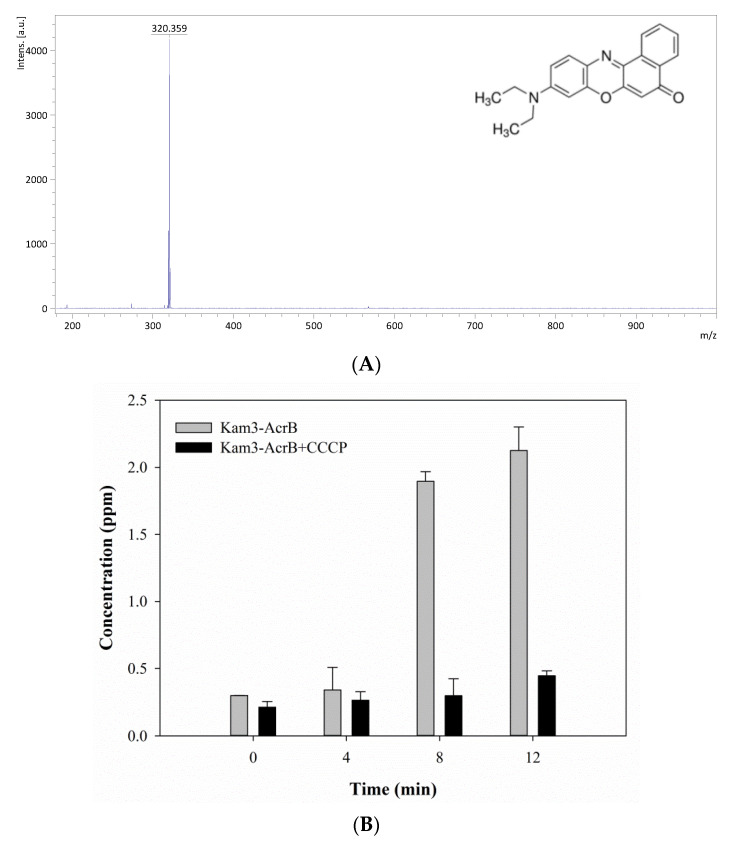
The Nile red efflux activity of Kam3-AcrB was detected by using MALDI-TOF MS in the presence of modulator CCCP. (**A**) The mass spectrum of Nile red and (**B**) the intensity of extracellular Nile red. The intensity was plotted at the main peak at *m*/*z* 320.36 of Nile red and the detection period was 12 min. Values are expressed as mean ± standard deviation (SD) (*n* = 3).

As shown in Figure 6A, the mass signal for Nile red was found at *m*/*z* 320.36. The Kam3-AcrB cells in the absence of CCCP increased the Nile red concentration from 0.30 ± 0.001 (*t* = 0) to 2.13 ± 0.17 ppm at 27 min in the extracellular space, and the concentrations of Nile red showed a range of 0.21 to 0.45 ppm for the cells added with CCCP (Figure 6B). Nile red functions as a fluorescent probe for intracellular lipids and hydrophobic domain proteins [43]. The rapid efflux of Nile red as shown in Kam3-AcrB in Figure 6B might be because lipophilic Nile red remains largely periplasmic and binds to membrane phospholipids, whereas AcrB collects its substrates from the periplasmic space and outer leaflet of the inner membrane. The presence of CCCP could abolish the transport of Nile red by AcrB, resulting in the intensity difference of extracellular Nile red between Kam3-AcrB and Kam-AcrB-CCCP in the reaction.

The data to monitor dye efflux by AcrB transporter using MALDI-TOF MS indicated continuous efflux of dyes over time in the energized Kam3-AcrB cells, and addition of inhibitor PAβN or CCCP abolished the efflux of dyes. These results were similar to the data in the dye accumulation and dye efflux assays, suggesting the reliability of determining dye efflux by drug transporter using MALDI-TOF MS. In addition, MALDI-TOF MS could detect dye efflux over time, possibly with little or no interference by substrates or inhibitors with strong colors, although the limitations for this method might be the need for a MALDI-TOF MS system along with additional sample preparation procedures, such as crystal formation of the tested samples.

### 2.5. Monitoring the Drug Efflux by Using MALDI-TOF

Although the determination of dye efflux over time by using MALDI-TOF indicated the efflux activity of multidrug transporter AcrB, it does not directly imply the drug efflux of AcrB in *E. coli*. Our previous IC_50_ data indicated that AcrB conferred resistance against erythromycin and rifampicin in the Kam3-AcrB cells, and addition of PAβN could reduce the IC_50_ for them. Thus, we further exploited MALDI-TOF to directly determine the efflux of antibiotics erythromycin and rifampicin by AcrB in *E. coli.*

Macrolides consist of a macrocyclic lactone of different ring sizes (usually 14-, 15- or 16-membered), to which one or more deoxy- or amino- sugar residues are attached [51]. Macrolides, such as erythromycin and clarithromycin, are used clinically as bacteriostatic antibiotics (might be bactericidal at higher concentrations) for treatment against infections by Gram-positive and limited Gram-negative bacteria, by binding to bacterial 50S ribosomal subunit and therefore interfering with protein synthesis [52]. Rifampicin inhibits bacterial RNA polymerase and is a potent bactericidal and broad-spectrum antibiotic to be used in clinical treatment against bacterial infections, such as tuberculosis [53]. The calibration curves of erythromycin (3.125 to 125 ppm) and rifampicin (0.5 to 20 ppm) were accomplished, where the *r*^2^ values were determined to be 0.9999 for all the calibration curves, representing a good correlation of intensity and drug concentration (Supplementary Figure S4).

**Figure 7:**
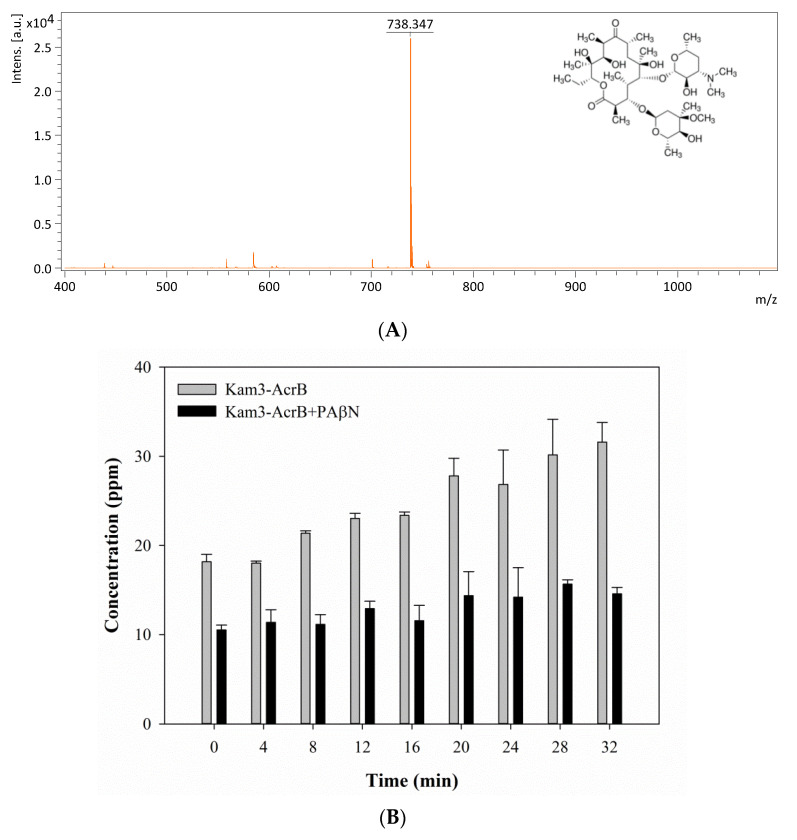
The erythromycin efflux activity of Kam3-AcrB was detected by using MALDI-TOF MS in the presence of modulator PAβN. (**A**) The mass spectrum of erythromycin and (**B**) the intensity of extracellular erythromycin. The intensity was plotted at the main peak at *m*/*z* 738.35 of erythromycin and the detection period was 32 min. Values are expressed as mean ± standard deviation (SD) (*n* = 3).

The mass spectrum of erythromycin is shown in Figure 7A and exhibits a main peak at *m*/*z* 738.34. The efflux of erythromycin by AcrB was measured by monitoring the intensity changes in extracellular erythromycin over time. The Kam3-AcrB cells in the absence of PAβN increased the erythromycin concentration from 18.18 ± 0.82 (*t* = 0) to 31.59 ± 2.20 ppm at 32 min in the extracellular space, and the increase in erythromycin was not observed in the cells added with PAβN (Figure 7B). These results demonstrated the continuous translocation of erythromycin, indicating the efflux-based resistance against erythromycin by AcrB.

**Figure 8:**
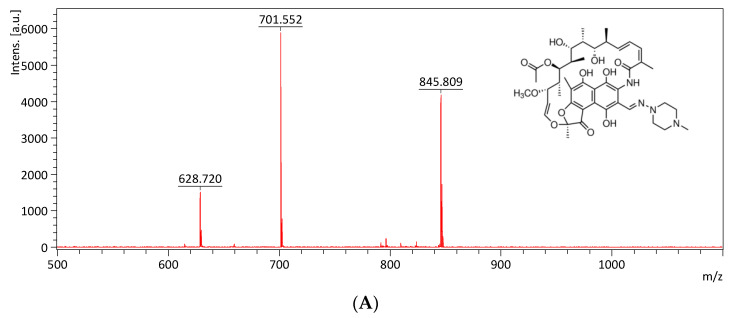

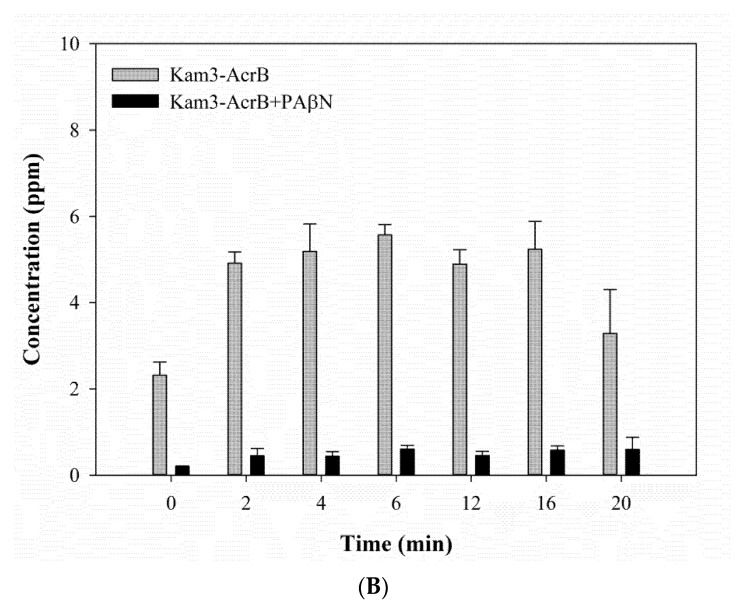
The rifampicin efflux activity of Kam3-AcrB was detected by using MALDI-TOF MS in the presence of modulator PAβN. (**A**) The mass spectrum of rifampicin and (**B**) the intensity of extracellular rifampicin. The intensity was plotted at the main peak at *m*/*z* 628.72, 701.55, and 845.81 of rifampicin and the detection period was 20 min. Values are expressed as mean ± standard deviation (SD) (*n* = 3).

The mass spectrum of rifampicin (monoisotopic mass 822.41) is shown in Figure 8A and exhibits a main peak at *m*/*z* 628.72, *m*/*z* 701.55 and *m*/*z* 845.81 (the sodium adduct of molecular ion peak). The Kam3-AcrB cells give an increase in the rifampicin concentration from 2.32 ± 0.30 (*t* = 0) to 5.24 ± 0.65 ppm at 16 min in the extracellular space; the rifampicin concentration was detected in a range of 0.21 to 0.60 ppm with the addition of PAβN for cells (Figure 8B). These results demonstrated the continuous translocation of rifampicin, indicating the efflux-based resistance against rifampicin by AcrB.

In this experiment, it was technically difficult to make the substrate intensity equal between the experimental and control group at time zero, especially for those substrates with rapid diffusion rate, since sample preparation needs 1 min centrifugation to remove the cells and obtain the supernatant (Section 3.7). During sample preparation, there was some diffusion and transport of the substrates across cell membranes in the limited time for sample preparation, and the efflux of the substrates was different between the experimental and control groups, causing the uneven substrate intensity in the extracellular space at time zero between them. It is quite intriguing that the greatest difference in the substrate intensity at time = 0 (min) between the control and experimental group was observed in the MS detection of extracellular rifampicin (Figure 8). It might be because PAβN possesses relatively greater inhibitory activity for the transport of rifampicin by AcrB, thus largely inhibiting the efflux of rifampicin during sample preparation. Our modulation data (Table 1) also indicated that PAβN showed a relatively greater modulation factor (MF = 64) for drug rifampicin.

The substrate concentrations used in MS analyses in this study were obtained from the concentration range of substrate calibration curves to obtain reliable results. The effective range for the tested compounds in this method could be related to the drug toxicity against the tested cells, influx/efflux of the compound, the compound ionization efficiency and sensitivity of the MS systems. The first trial of drug concentration to be used in sample preparation for MS analysis is suggested to be its sub-MIC against the tested cells. This could possibly give reasonable results provided that the substrate concentrations in the extracellular space during the experiment are within the concentration range of its calibration curve.

High-performance LC–electrospray ionization–MS (LC-ESI-MS) has been reported to determine the intracellular concentration of drugs for the detection of drug efflux. The accumulation of fleroxacin and ciprofloxacin in the *E*. *coli* cells was found to be less in those cells overexpressing drug transporters [54]. Chu et al. [55] indicated that intracellular concentrations of drugs or metabolites should be known in order to determine drug interaction and toxicity, but detection of the intracellular concentrations of chemicals might be interfered with by the cell lysis efficacy and the extraction step. Pitt [56] indicated that LC-MS provides great resolution and precise quantitative analysis, so it was used to determine the contents of diverse samples, such as wastes, metabolites and proteins; however, the time needed for analysis of each sample might limit the number of samples. This study used MALDI-TOF MS to measure the drug efflux in cell supernatant without the cell extraction steps and to prevent the loss of drugs during the experimental process, providing an easy, rapid and reliable method to determine the drug efflux in the presence of the transporter.

## 3. Materials and Methods

### 3.1. Strains and Plasmids

The *E. coli* strain Novablue (DE3) was used for genetic manipulation. Drug susceptible *E. coli* strain Kam3, an *acrB* deletion strain, was used for AcrB overexpression, drug resistance assays, drug accumulation tests and drug efflux tests [57]. The pSYC vectors were used for *acrB* gene cloning and overexpression.

### 3.2. Construction of Kam3 Harboring pSYC-acrB Plasmid

*E*. *coli* strain Novablue, derived from *E*. *coli* K12, served as the source of chromosomal DNA for the *acrB* genes. The complete genome of *E*. *coli* strain K-12 substrain MG1655 can be found in the NCBI database (RefSeq NC_000913.3), which is a source of sequence information. *acrB* gene was cloned from the *E. coli* K12 chromosome by using PCR method. The *acrB* gene was amplified by using primers 5′-AAAACCCATATGCCTAATTTCTTTATCGATCGCC-3′ and 5′-AAAACCGTCGACTCAATGATGATCGACAGTAT-3′, which was digested with *NdeI* and *SalI* restriction enzymes and inserted into pSYC vector at the *NdeI*-*SalI* site. The pSYC plasmid encoding *acrB* was transformed into drug susceptible *E. coli* strain Kam3 for protein overexpression, drug susceptibility test, dye accumulation assay, dye efflux assay and drug efflux assay.

### 3.3. Overexpression, Purification and Identification of Multidrug Transporter AcrB

*E. coli* Kam3 harboring pSYC plasmid encoding AcrB was cultivated at 37 °C, induced with 0.2 mM IPTG and overexpressed at 24 °C overnight. The cells were collected by centrifugation and disrupted by using French press. The pellet was removed by centrifugation, and the supernatant was collected. The supernatant was centrifuged at 43,000 rpm for 120 min to spin down the cell membranes, which were collected and solubilized in 2% (*v*/*v*) Triton X-100. Detergent solubilized AcrB was purified by using nickel affinity column (Hitrap chelating column, GE) and identified by peptide mass fingerprinting.

### 3.4. Broth Dilution Assay

The IC_50_ of various antimicrobials to *E. coli* overexpressing AcrB was determined by the broth dilution method [58]. Briefly, IC_50_ was determined by incubating the cells at log phase (OD600 = 0.6–0.8) and suspending them with Muller–Hinton broth to obtain a density of 10^5^ cfu/mL. Each drug was diluted with Muller–Hinton broth in a two-fold serial dilution series in a flat-bottomed 96-well plate. The cells were incubated at 37 °C for 12 h, and cell growth was examined using spectroscopy to determine the IC_50_ of antimicrobial drugs.

### 3.5. Dye Accumulation Assay

The dye accumulation assay was carried out as previously described, with the following modifications [17]. The *E. coli* cells were incubated to OD600 of 0.6 to 0.8 in MH broth and collected by centrifugation (5000 rpm, 5 min and 4 °C). The cells were resuspended twice in PBS buffer and diluted in PBS in a final OD600 of 0.6. The cell suspension was incubated in 96-well plates with the filter-sterilized glucose to a final concentration of 25 mM at room temperature for 3 min. The dye H33342 and EtBr was added with glucose to a final concentration of 1 and 25 μM, respectively. The fluorescence was measured over 38 min for dye accumulation measurement (Ex 360 nm and Em 460 nm for H33342; Ex 520 nm and Em 600 nm for EtBr) [17,43]. The efflux pump inhibitors of PAβN and CCCP were used to a final concentration of 20 μg/mL.

### 3.6. Dye Efflux Assay

The dye efflux assay was carried out as previously described, with the following modifications [43]. The *E. coli* cells were incubated to OD600 of 0.6 to 0.8 in MH broth and collected by centrifugation (5000 rpm, 5 min and 4 °C). The cells were resuspended twice in PBS buffer and diluted in PBS in a final OD600 of 0.6. The cell suspension was incubated with dye H33342, EtBr and Nile red to a final concentration of 1, 25 and 5 μM, respectively, and incubated at room temperature for 8 h. The cells were resuspended twice in PBS buffer and incubated in 96-well plates with the filter-sterilized glucose to a final concentration of 25 mM at room temperature. The fluorescence was measured over 38 min for dye efflux measurement (Ex 360 nm and Em 460 nm for H33342; Ex 520 nm and Em 600 nm for EtBr; Ex 550 nm and Em 650 nm for Nile red). The t_efflux50%_ is the time required for cells to extrude 50% of preloaded dye, the efflux rate constant (K value). EtBr efflux curves were fitted using a single exponential decay equation as follows: Y = (Y_0_ − Plateau) × exp(−K × X) + Plateau, where X is the time, Y is the EtBr efflux that starts at Y_0_ and decays to the plateau in one phase; the plateau is Y at infinite times. The plateau has the same units as Y and the K or slope is the efflux rate constant [59].

### 3.7. Sample Preparation and Mass Spectrometry Analysis

To generate the standard curves, various concentrations of the dyes (H33342, EtBr and Nile red) and drugs (erythromycin and rifampicin) were dissolved in ammonium bicarbonate buffer (ABC buffer, 10 mM), respectively, and mixed with matrix 2,5-dihydroxybenzoic acid (DHB) for MALDI-TOF MS analysis (Autoflex III, Bruker Daltonics, Bilerica, MA, USA). The *E. coli* cells were incubated at 37 °C to an OD600 of 0.6 to 0.8 in MH broth and collected by centrifugation (6000× *g* for 5 min at 4 °C). The cells were resuspended twice and diluted in ABC buffer (pH = 7.5) to a final OD600 of 0.6. To monitor the efflux efficiency (drug or dye) by transporter AcrB using MALDI-TOF, the cells harboring pSYC plasmid encoding AcrB were added with a substrate (H33342/EtBr/Nile red/erythromycin/rifampicin to a final concentration of 10/10/30/125/10 ppm) and filter-sterilized glucose (50 mM), and the samples were taken from the cell mixtures at various time points until the end of reaction. Once collected, the sample at each time point was centrifuged (6000× *g* for 1 min) to obtain the supernatant for MS analysis. The samples were mixed with DHB matrix and analyzed by using MALDI-TOF MS. Data acquisition was performed automatically (random walk mode) in steps of 50 shots for a total of 10,000 shots per sample. Mass spectra were analyzed by FlexAnalysis (version 3.0, Bruker Daltonics) and the ion abundancy of substrates (H33342, EtBr, Nile red, erythromycin or rifampicin) from each spot was derived by the integration of signal. 

### 3.8. Statistics Analysis

Data were analyzed statistically using SPSS Version 12.0 (SPSS Inc., Chicago, IL, USA). One-way analysis of variance (ANOVA) was used to determine statistical differences between sample means, with the level of significance set at *p* < 0.05. Multiple comparisons of means were done by Duncan’s test. All data are expressed as mean ± SD.

## 4. Conclusions

The use of MALDI-TOF MS for drug efflux determination provides the advantages of fewer sample preparation steps, rapid manipulation and reliable analysis. This study found that MALDI-TOF MS could directly determine the amount of drug and continuously monitor the efflux of the drug transporter. The substrates were collected in the extracellular space at different time points, and their intensities were analyzed by MALDI-TOF MS so as to indicate the degree of substrate efflux over time. This method should be not only valuable for a direct determination of efflux-based antibiotic resistance and for drugs that are not intrinsically fluorescent or additionally labeled with radiolabels but also for the evaluation of efflux inhibitory activity of colorful crude extracts and compounds. 

## Figures and Tables

**Table 1:** The IC_50_ of Kam3-AcrB against drugs and dyes in the presence of modulator PAβN and CCCP.

Drugs and Dyes	Kam3-AcrB IC_50_ (μg/mL)	Kam3-AcrB + PAβN		Kam3-AcrB + CCCP	
		IC_50_ (μg/mL)	MF	IC_50_ (μg/mL)	MF
**Macrolide**					
Erythromycin	250	31.25	8	31.25	8
Clarithromycin	87.5	21.87	4	3.5	25
**Quinolone**					
Norfloxacin	1.56	1.56	1	0.015	100
**Tetracycline**					
Tetracycline	0.39	0.16	2	0.16	2
Rifampicin	1.25	0.019	64	1.25	1
**Dyes**					
Hoechst 33342	15.625	7.815	2	0.488	32
Ethidium bromide	100	50	2	25	4
Nile red	>16	>16	1	>16	1

MF: modulation factor.

## Supplementary Materials

The following are available online at https://www.mdpi.com/2079-6382/9/10/639/s1, Figure S1: Identification of the purified AcrB protein, Figure S2: The influx and efflux of the substrates when they are incubated with the *E. coli* cells, Figure S3: The calibration curves of dyes, Figure S4. The calibration curves of drugs, Table S1: The IC_50_ of Kam3, and Kam3-AcrB against dyes and drugs.

## References

[1] Lu W.J., Lin H.J., Janganan T.K., Li C.Y., Chin W.C., Bavro V.N., Lin H.T.V. (2018). ATP-Binding Cassette Transporter VcaM from *Vibrio cholerae* is Dependent on the Outer Membrane Factor Family for Its Function. Int. J. Mol. Sci..

[2] Rees D.C., Johnson E., Lewinson O. (2009). ABC transporters: The power to change. Nat. Rev. Mol. Cell Biol..

[3] Kourtesi C., Ball A.R., Huang Y.Y., Jachak S.M., Vera D.M., Khondkar P., Gibbons S., Hamblin M.R., Tegos G.P. (2013). Microbial efflux systems and inhibitors: Approaches to drug discovery and the challenge of clinical implementation. Open Microbiol. J..

[4] Piddock L.J.V. (2006). Multidrug-resistance efflux pumps-not just for resistance. Nat. Rev. Microbiol..

[5] Nikaido H. (1996). Multidrug efflux pumps of gram-negative bacteria. J. Bacteriol..

[6] Blair J.M.A., Piddock L.J.V. (2016). How to measure export via bacterial multidrug resistance efflux pumps. Mbio.

[7] Iqbal J., Siddiqui R., Kazmi S.U., Khan N.A. (2013). A simple assay to screen antimicrobial compounds potentiating the activity of current antibiotics. BioMed Res. Int..

[8] Otrebska-Machaj E., Chevalier J., Handzlik J., Szymanska E., Schabikowski J., Boyer G., Bolla J.M., Kiec-Kononowicz K., Pages J.M., Alibert S. (2016). Efflux Pump Blockers in Gram-Negative Bacteria: The New Generation of Hydantoin Based-Modulators to Improve Antibiotic Activity. Front. Microbiol..

[9] Pumbwe L., Piddock L.J. (2002). Identification and molecular characterisation of CmeB, a *Campylobacter jejuni* multidrug efflux pump. FEMS Microbiol. Lett..

[10] Baucheron S., Imberechts H., Chaslus-Dancla E., Cloeckaert A. (2002). The AcrB multidrug transporter plays a major role in high-level fluoroquinolone resistance in Salmonella enterica serovar typhimurium phage type DT204. Microb. Drug Resist..

[11] Kern W.V., Steinke P., Schumacher A., Schuster S., von Baum H., Bohnert J.A. (2006). Effect of 1-(1-naphthylmethyl)-piperazine, a novel putative efflux pump inhibitor, on antimicrobial drug susceptibility in clinical isolates of *Escherichia coli*. J. Antimicrob. Chemother..

[12] Lomovskaya O., Warren M.S., Lee A., Galazzo J., Fronko R., Lee M., Blais J., Cho D., Chamberland S., Renau T. (2001). Identification and characterization of inhibitors of multidrug resistance efflux pumps in *Pseudomonas aeruginosa*: Novel agents for combination therapy. Antimicrob. Agents Chemother..

[13] Kaatz G.W., Moudgal V.V., Seo S.M., Kristiansen J.E. (2003). Phenothiazines and thioxanthenes inhibit multidrug efflux pump activity in *Staphylococcus aureus*. Antimicrob. Agents Chemother..

[14] Iyer R., Erwin A.L. (2015). Direct measurement of efflux in *Pseudomonas aeruginosa* using an environment-sensitive fluorescent dye. Res. Microbiol..

[15] Kobayashi N., Nishino K., Yamaguchi A. (2001). Novel macrolide-specific ABC-type efflux transporter in *Escherichia coli*. J. Bacteriol..

[16] Paixao L., Rodrigues L., Couto I., Martins M., Fernandes P., de Carvalho C.C., Monteiro G.A., Sansonetty F., Amaral L., Viveiros M. (2009). Fluorometric determination of ethidium bromide efflux kinetics in *Escherichia coli*. J. Biol. Eng..

[17] Richmond G.E., Chua K.L., Piddock L.J.V. (2013). Efflux in *Acinetobacter baumannii* can be determined by measuring accumulation of H33342 (bis-benzamide). J. Antimicrob. Chemother..

[18] Misra R., Morrison K.D., Cho H.J., Khuu T. (2015). Importance of real-time assays to distinguish multidrug efflux pump-inhibiting and outer membrane-destabilizing activities in *Escherichia coli*. J. Bacteriol..

[19] Machado D., Perdigão J., Portugal I., Pieroni M., Silva P., Couto I., Viveiros M. (2018). Efflux activity differentially modulates the levels of isoniazid and rifampicin resistance among multidrug resistant and monoresistant *Mycobacterium tuberculosis* strains. Antibiotics.

[20] Coldham N.G., Webber M., Woodward M.J., Piddock L.J. (2010). A 96-well plate fluorescence assay for assessment of cellular permeability and active efflux in *Salmonella enterica* serovar Typhimurium and *Escherichia coli*. J. Antimicrob. Chemother..

[21] Shapiro A.B., Corder A.B., Ling V. (1997). P-glycoprotein-mediated Hoechst 33342 transport out of the lipid bilayer. Eur. J. Biochem..

[22] Babayan A., Nikaido H. (2004). In *Pseudomonas aeruginosa* ethidium bromide does not induce its own degradation or the assembly of pumps involved in its efflux. Biochem. Biophys. Res. Commun..

[23] Howitz K.T., Bitterman K.J., Cohen H.Y., Lamming D.W., Lavu S., Wood J.G., Zipkin R.E., Chung P., Kisielewski A., Zhang L.L. (2003). Small molecule activators of sirtuins extend *Saccharomyces cerevisiae* lifespan. Nature.

[24] Brown A.R., Ettefagh K.A., Todd D., Cole P.S., Egan J.M., Foil D.H., Graf T.N., Schindler B.D., Kaatz G.W., Cech N.B. (2015). A mass spectrometry-based assay for improved quantitative measurements of efflux pump inhibition. PLoS ONE.

[25] Hrabak J., Bitar I., Papagiannitsis C.C. (2019). Combination of mass spectrometry and DNA sequencing for detection of antibiotic resistance in diagnostic laboratories. Folia Microbiol..

[26] Murakami S., Nakashima R., Yamashita E., Yamaguchi A. (2002). Crystal structure of bacterial multidrug efflux transporter AcrB. Nature.

[27] Kobylka J., Kuth M.S., Muller R.T., Geertsma E.R., Pos K.M. (2019). AcrB: A mean, keen, drug efflux machine. Ann. N. Y. Acad. Sci..

[28] Costa S.S., Viveiros M., Amaral L., Couto I. (2013). Multidrug Efflux Pumps in *Staphylococcus aureus*: An Update. Open Microbiol. J..

[29] Opperman T.J., Nguyen S. (2015). Recent advances toward a molecular mechanism of efflux pump inhibition. Front. Microbiol..

[30] Opperman T.J., Kwasny S.M., Kim H.S., Nguyen S.T., Houseweart C., D’Souza S., Walker G.C., Peet N.P., Nikaido H., Bowlin T.L. (2014). Characterization of a novel pyranopyridine inhibitor of the AcrAB efflux pump of *Escherichia coli*. Antimicrob. Agents Chemother..

[31] Soto S.M., Ruiz J., Mendoza M.C., Vila J. (2003). In vitro fluoroquinolone-resistant mutants of *Salmonella enterica* serotype Enteritidis: Analysis of mechanisms involved in resistance. Int. J. Antimicrob. Agents.

[32] Grimsey E.M., Fais C., Marshall R.L., Ricci V., Ciusa M.L., Stone J.W., Ivens A., Malloci G., Ruggerone P., Vargiu A.V. (2020). Chlorpromazine and Amitriptyline Are Substrates and Inhibitors of the AcrB Multidrug Efflux Pump. MBio.

[33] Takatsuka Y., Chen C., Nikaido H. (2010). Mechanism of recognition of compounds of diverse structures by the multidrug efflux pump AcrB of *Escherichia coli*. Proc. Natl. Acad. Sci. USA.

[34] Nakashima R., Sakurai K., Yamasaki S., Nishino K., Yamaguchi A. (2011). Structures of the multidrug exporter AcrB reveal a proximal multisite drug-binding pocket. Nature.

[35] Li G., Zhang J., Guo Q., Wei J., Jiang Y., Zhao X., Zhao L.L., Liu Z., Lu J., Wan K. (2015). Study of efflux pump gene expression in rifampicin-monoresistant *Mycobacterium tuberculosis* clinical isolates. J. Antibiot..

[36] Van den Berg van Saparoea H.B., Lubelski J., van Merkerk R., Mazurkiewicz P.S., Driessen A.J. (2005). Proton motive force-dependent Hoechst 33342 transport by the ABC transporter LmrA of *Lactococcus lactis*. Biochemistry.

[37] Whittle E.E., Legood S.W., Alav I., Dulyayangkul P., Overton T.W., Blair J.M.A. (2019). Flow cytometric analysis of efflux by dye accumulation. Front. Microbiol..

[38] Morgan A.R., Evans D.H., Lee J.S., Pulleyblank D.E. (1979). Review: Ethidium fluorescence assay. Part II. Enzymatic studies and DNA-protein interactions. Nucleic Acids Res..

[39] Cosa G., Focsaneanu K.S., McLean J.R., McNamee J.P., Scaiano J.C. (2001). Photophysical properties of fluorescent DNA-dyes bound to single- and double-stranded DNA in aqueous buffered solution. Photochem. Photobiol..

[40] Sharma A., Gupta V.K., Pathania R. (2019). Efflux pump inhibitors for bacterial pathogens: From bench to bedside. Indian J. Med. Res..

[41] Kinana A.D., Vargiu A.V., May T., Nikaido H. (2016). Aminoacyl beta-naphthylamides as substrates and modulators of AcrB multidrug efflux pump. Proc. Natl. Acad. Sci. USA.

[42] Dreier J., Ruggerone P. (2015). Interaction of antibacterial compounds with RND efflux pumps in *Pseudomonas aeruginosa*. Front. Microbiol..

[43] Bohnert J.A., Karamian B., Nikaido H. (2010). Optimized Nile Red Efflux Assay of AcrAB-TolC Multidrug Efflux System Shows Competition between Substrates. Antimicrob. Agents Chemother..

[44] Bucevičius J., Lukinavičius G., Gerasimaitė R. (2018). The use of hoechst dyes for DNA staining and beyond. Chemosensors.

[45] Li X.L., Hu Y.J., Wang H., Yu B.Q., Yue H.L. (2012). Molecular Spectroscopy Evidence of Berberine Binding to DNA: Comparative Binding and Thermodynamic Profile of Intercalation. Biomacromolecules.

[46] Dos Santos J.F.S., Tintino S.R., de Freitas T.S., Campina F.F., Irwin R.D.A., Siqueira-Junior J.P., Coutinho H.D.M., Cunha F.A.B. (2018). In vitro e in silico evaluation of the inhibition of *Staphylococcus aureus* efflux pumps by caffeic and gallic acid. Comp. Immunol. Microbiol. Infect. Dis..

[47] Raherison S., Gonzalez P., Renaudin H., Charron A., Bebear C., Bebear C.M. (2002). Evidence of active efflux in resistance to ciprofloxacin and to ethidium bromide by *Mycoplasma hominis*. Antimicrob. Agents Chemother..

[48] Nikaido H., Thanassi D.G. (1993). Penetration of lipophilic agents with multiple protonation sites into bacterial cells: Tetracyclines and fluoroquinolones as examples. Antimicrob. Agents Chemother..

[49] Mortimer P.G., Piddock L.J. (1991). A comparison of methods used for measuring the accumulation of quinolones by Enterobacteriaceae, *Pseudomonas aeruginosa* and *Staphylococcus aureus*. J. Antimicrob. Chemother..

[50] Cinquin B., Maigre L., Pinet E., Chevalier J., Stavenger R.A., Mills S., Refregiers M., Pages J.M. (2015). Microspectrometric insights on the uptake of antibiotics at the single bacterial cell level. Sci. Rep..

[51] Mazzei T., Mini E., Novelli A., Periti P. (1993). Chemistry and mode of action of macrolides. J. Antimicrob. Chemother..

[52] Leclercq R. (2002). Mechanisms of resistance to macrolides and lincosamides: Nature of the resistance elements and their clinical implications. Clin. Infect. Dis..

[53] Campbell E.A., Korzheva N., Mustaev A., Murakami K., Nair S., Goldfarb A., Darst S.A. (2001). Structural mechanism for rifampicin inhibition of bacterial RNA polymerase. Cell.

[54] Dumont E., Vergalli J., Conraux L., Taillier C., Vassort A., Pajovic J., Refregiers M., Mourez M., Pages J.M. (2019). Antibiotics and efflux: Combined spectrofluorimetry and mass spectrometry to evaluate the involvement of concentration and efflux activity in antibiotic intracellular accumulation. J. Antimicrob. Chemother..

[55] Chu X., Korzekwa K., Elsby R., Fenner K., Galetin A., Lai Y., Matsson P., Moss A., Nagar S., Rosania G.R. (2013). Intracellular Drug Concentrations and Transporters: Measurement, Modeling, and Implications for the Liver. Clin. Pharm..

[56] Pitt J.J. (2009). Principles and applications of liquid chromatography-mass spectrometry in clinical biochemistry. Clin. Biochem. Rev..

[57] Morita Y., Kodama K., Shiota S., Mine T., Kataoka A., Mizushima T., Tsuchiya T. (1998). NorM, a putative multidrug efflux protein, of *Vibrio parahaemolyticus* and its homolog in *Escherichia coli*. Antimicrob. Agents Chemother..

[58] Lu W.J., Lin H.J., Hsu P.H., Lai M., Chiu J.Y., Lin H.T.V. (2019). Brown and red seaweeds serve as potential efflux pump inhibitors for drug-resistant *Escherichia coli*. Evid.-Based Complement. Altern. Med..

[59] Iyer R., Ferrari A., Rijnbrand R., Erwin A.L. (2015). A Fluorescent Microplate Assay Quantifies Bacterial Efflux and Demonstrates Two Distinct Compound Binding Sites in AcrB. Antimicrob. Agents Chemother..

