# Supplementary material for: Determination of Drug Efflux Pump Efficiency in Drug-Resistant Bacteria Using MALDI-TOF MS
Source: Antibiotics (Basel). 2020 Sep 24;9(10):639. doi: 10.3390/antibiotics9100639 (PMC7598683; doi:10.3390/antibiotics9100639)
Supplement: Supplementary file 1 [file antibiotics-09-00639-s001.pdf]

(A)

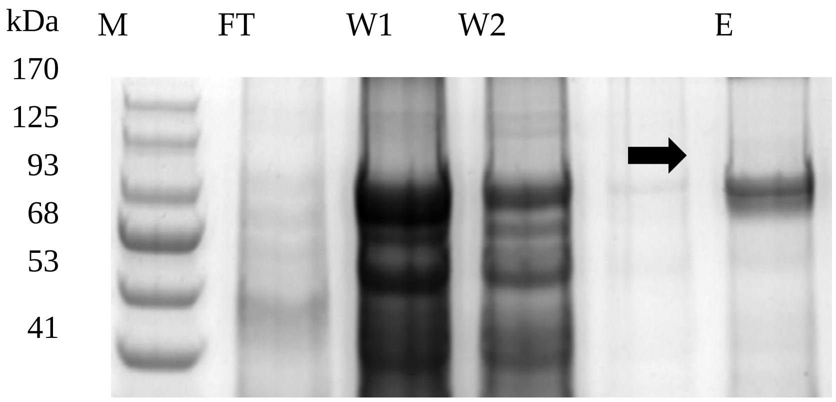

(B)

```
   1 MPNFFIDRPI FAWVIAIIIM LAGGLAILKL PVAQYPTIAP PAVTISASYP
  51 GADAKTVQDT VTQVIEQNMN GIDNLMYMSS NSDSTGTVQI TLTFESGTDA
 101 DIAQVQVQNK LQLAMPLLPQ EVQQQGVSVE KSSSSFLMVV GVINTDGTMT
 151 QEDISDYVAA NMKDAISRTS GVGDVQLFGS QYAMRIWMNP NELNKFQLTP
 201 VDVITAIKAQ NAQVAAGQLG GTPPVKGQQL NASIIAQTRL TSTEEFGKIL
 251 LKVNQDGSRV LLRDVAKIEL GGENYDIIAE FNGQPASGLG IKLATGANAL
 301 DTAAAIRAEL AKMEPFFPSG LKIVYPYDTT PFVKISIHEV VKTLVEAIIL
 351 VFLVMYLFLQ NFRATLIPTI AVPVVLLGTF AVLAAFGFSI NTLTMFGMVL
 401 AIGLLVDDAI VVVENVERVM AEEGLPPKEA TRKSMGQIQG ALVGIAMVLS
 451 AVFVPMAFFG GSTGAIYRQF SITIVSAMAL SVLVALILTP ALCATMLKPI
 501 AKGDHGEGKK GFFGWFNRMF EKSTHHYTDS VGGILRSTGR YLVLYLIIVV
 551 GMAYLFVRLP SSFLPDEDQG VFMTMVQLPA GATQERTQKV LNEVTHYYLT
 601 KEKNNVESVF AVNGFGFAGR GQNTGIAFVS LKDWADRPGE ENKVEAITMR
 651 ATRAFSQIKD AMVFAFNLPA IVELGTATGF DFELIDQAGL GHEKLTQARN
 701 QLLAEAAKHP DMLTSVRPNG LEDTPQFKID IDQEKAQALG VSINDINTTL
 751 GAAWGGSYVN DFIDRGRVKK VYVMSEAKYR MLPDDIGDWY VRAADGQMVP
 801 FSAFSSSRWE YGSPRLERYN GLPSMEILGQ AAPGKSTGEA MELMEQLASK
 851 LPTGVGYDWT GMSYQERLSG NQAPSLYAIS LIVVFLCLAA LYESWSIPFS
 901 VMLVVPLGVI GALLAATFRG LTNDVYFQVG LLTTIGLSAK NAILIVEFAK
 951 DLMDKEGKGL IEATLDAVRM RLRPILMTSL AFILGVMPLV ISTGAGSGAQ
1001 NAVGTGVMGG MVTATVLAIF FVPVFFVVVR RRFSRKNEDI EHSHTVDHH
```

Figure 1. Identification of the purified AcrB protein. (A) SDS-PAGE. M: protein molecular weight marker; FT: flow through; W1: wash by 30 mM imidazole; W2: wash by 50mM imidazole; E: elution by 500 mM imidazole. The black arrow indicated overexpressed AcrB. **(B) AcrB sequence coverage determined by MS.** Taxonomy: *E. coli* K-12. Sequence coverage: 41%. Identified peptides were shown in red.

Table S1. The IC_{50} of Kam3, and Kam3-AcrB against dyes and drugs.

**Drug group and drug**	**IC_{50} (μg/mL)**		**Relative resistance**
	Kam3	Kam3-AcrB	
	**Macrolide**		
Erythromycin	7.812	250	32
Clarithromycin	10.937	87.5	8
	**Quinolone**		
Norfloxacin	0.78	1.56	2
	**Tetracycline**		
Tetracycline	0.098	0.39	4
**Rifampicin**	0.625	1.25	2
**Dyes**			
Hoechst 33342	7.812	15.625	2
Ethidium bromide	25	100	4
Nile red	>16	>16	1

(C)

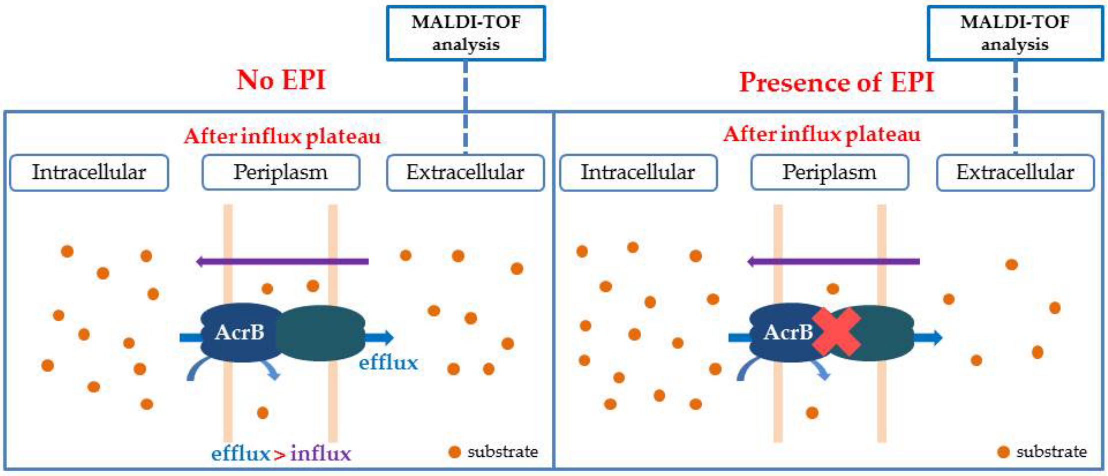

Increased substrates in the extracellular space monitored by using MALDI-TOF.

Fewer substrates in the extracellular space as compared with the no EPI group.

(A)

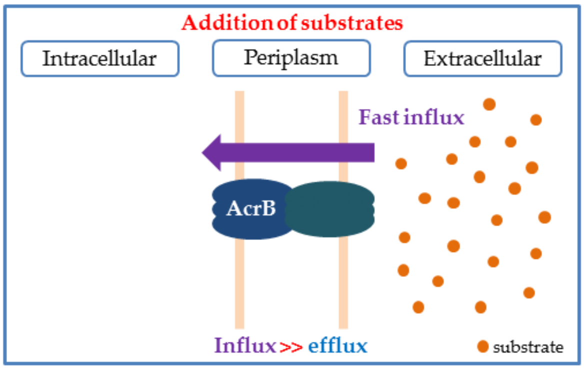

At time = 0, substrates started to get across the cell membrane by passive diffusion. The influx rate was much greater than the efflux rate.

(B)

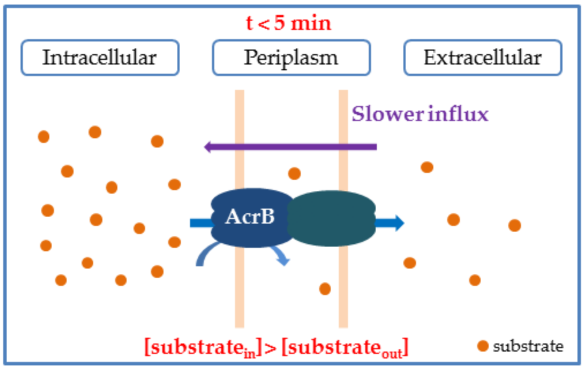

Substrates influx gradually reached a plateau within 5 min.

Figure S2. The influx and efflux of the substrates when they are incubated with the *E. coli* cells.

(A)

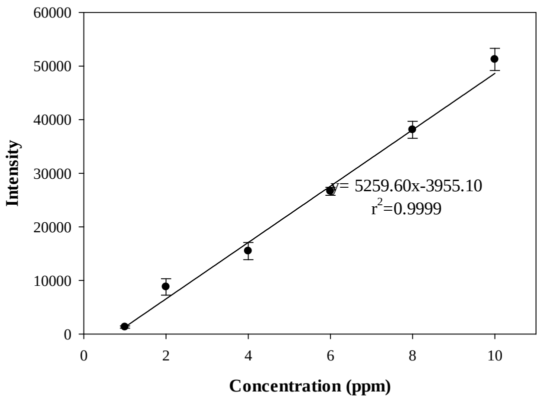

(B)

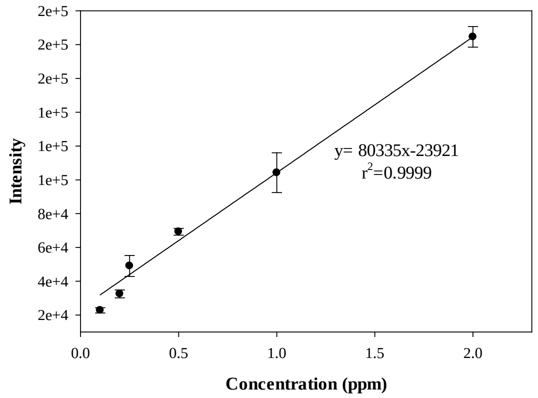

(C)

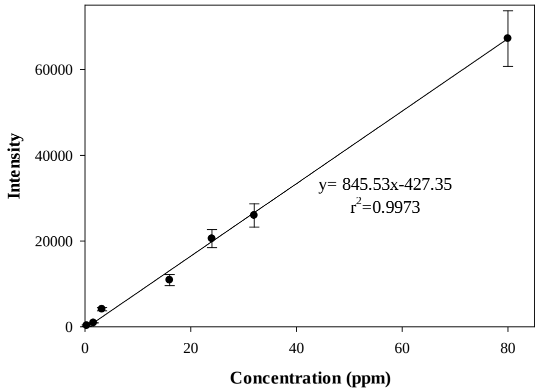

Figure S3. The calibration curves of dyes (A) Hoechst 33342 (B) EtBr (C) Nile red. Values were expressed as mean ± standard deviation (SD) (n = 3).

(A)

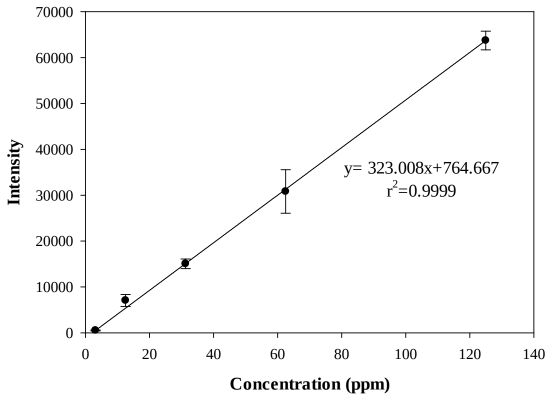

(B)

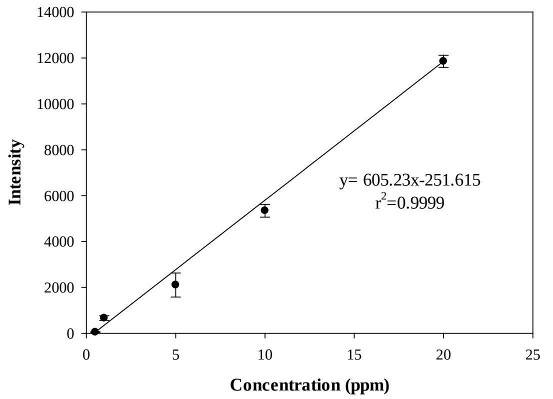

Figure S4. The calibration curves of dyes (**A**) Erythromycin (**B**) Rifampicin. Values were expressed as mean ± standard deviation (SD) (n = 3).
